# Comparison of Volumetric Modulated Arc Therapy (VMAT) and Conventional Intensity-Modulated Radiotherapy (IMRT) for Locally Advanced Head and Neck Squamous Cell Carcinoma: A Retrospective Cohort Study

**DOI:** 10.7759/cureus.61022

**Published:** 2024-05-24

**Authors:** Arash Algouneh, Ken Schneider, Kitty Huang, Abdulkadir Hussein, Ming Pan

**Affiliations:** 1 Radiation Oncology, Schulich School of Medicine and Dentistry, London, CAN; 2 Radiation Oncology, Windsor Regional Hospital Cancer Program, Windsor, CAN; 3 Mathematics amd Statistics, University of Windsor, Windsor, CAN

**Keywords:** overall survival, locoregional control, vmat, cimrt, hpv-related hnscc, hnscc treatment

## Abstract

Purpose

This study examines the outcomes of locally advanced head and neck squamous cell carcinoma (HNSCC) following the adoption of conventional intensity-modulated radiotherapy (cIMRT) and volumetric-modulated arc therapy (VMAT) over a decade. The region under study has higher comorbidities associated with increased HNSCC incidence and poorer prognosis.

Materials and methods

A 10-year retrospective review of electronic medical records included 296 patients with stage III, IVA, and IVB HNSCC (American Joint Committee on Cancer, Seventh edition). Survival outcomes were compared between VMAT and cIMRT using Kaplan-Meier survival curves and adjusted for relevant demographic factors using Cox's proportional hazards model. Analysis was performed using R software (R Foundation, Vienna, Austria).

Results

The median age of the cohort was 63 years, comprising of 80% males. The oropharynx was the most common primary tumor site. 264 (89%) received 50Gy or higher dose radiation by either cIMRT (22%) or VMAT (67%). At five years, locoregional control (LC) and overall survival (OS) rates were 79.5% and 56.7%, respectively. VMAT showed a significant improvement in five-year OS (63.4% versus 43.8% for cIMRT, p=0.0023) but no significant difference in five-year LC (81% VMAT versus 74.5% cIMRT, p=0.17). Grade 3-4 acute toxicity was observed in 22% of patients.

Conclusions

VMAT and cIMRT demonstrated excellent LC in locally advanced HNSCC despite high comorbidity rates. Notably, VMAT was associated with significantly better OS compared to cIMRT. These outcomes surpass historical data, suggesting that VMAT technology may lead to improved patient outcomes. However, larger randomized controlled trials and dosimetric studies are needed to confirm these findings.

## Introduction

In 2022, approximately 7500 Canadians were diagnosed with head and neck cancer, and 2100 Canadians died from this disease. The most common head and neck malignancy (up to 90%) is head and neck squamous cell carcinoma (HNSCC), which develops from the mucosal epithelium in the oral cavity, pharynx, and larynx [1]. Some risk factors for HNSCC include tobacco and alcohol consumption, exposure to environmental pollutants, and infection with viral agents. Specifically, persistent infection with human papillomavirus (HPV) and Epstein-Barr virus (EBV) are known etiological risk factors for HNSCC arising from the oropharynx and nasopharynx, respectively [2,3].

The diagnosis of HNSCC is established by biopsy of the primary tumor [4]. Additionally, since patients with HPV-positive oropharyngeal cancer have consistently better survival than those with HPV-negative oropharyngeal cancer, HPV testing by immunohistochemistry for p16, a tumor suppressor, has become common for oropharyngeal or primary unknown cancer of the head and neck region [5,6]. The anatomical site of the disease, stage, functional considerations, and patient preferences determine the treatment approach.

Radiation therapy is an effective treatment for HNSCC as a primary modality, specifically in advanced HNSCC, and as an adjuvant treatment following surgery [1]. However, radiation therapy is associated with acute and chronic toxicities such as mucositis, dysphagia, xerostomia, dermatitis, and pain [7,8]. Specifically, late radiation toxicity is permanent and results in reduced quality of life (QOL) for the patient, particularly xerostomia and dysphagia [9]. As a result, radiation therapy for advanced HNSCC has shifted from three-dimensional conformal radiotherapy (3D-CRT) to intensity-modulated radiotherapy (IMRT). IMRT has reduced toxicities, specifically the sparing of the parotid gland, resulting in the decrease of xerostomia for patients treated with IMRT compared with 3D-CRT [7,10]. IMRT, as a definitive treatment for HNSCC, has become the standard of care in many institutions worldwide [11]. Volumetric modulated arc therapy (VMAT) is a newer technique of IMRT that delivers radiation dose using a rotating gantry with varying speed and dose rate in contrast to conventional IMRT (cIMRT), which uses fixed gantry beams. Although several studies have demonstrated shorter planning and treatment time, improved dose homogeneity, and normal tissue sparing when comparing VMAT to cIMRT, few studies have explored its long-term clinical outcomes [12,13].

Our cancer center serves a population base of approximately 650,000. Higher smoking rates [14] and alcohol consumption than the provincial average [15] and the low HPV vaccination rates [16] could ultimately be contributing factors to higher incidence and poorer prognosis of HNSCC in our region. We started cIMRT in 2009 and VMAT in 2012 as the standard radiation modality for the definitive treatment of locally advanced HNSCC. This study aims to review our experience with stage III, IVA, and IVB HNSCC treated with VMAT versus cIMRT by comparing outcomes and the toxicity of these two modalities over a decade.

## Materials and methods

Study approval and patient selection

This retrospective study received approval from the Windsor Regional Cancer Center's Research Ethics Board (REB) (WRH REB #21-396). We reviewed the electronic medical records (EMR) of patients presenting with head and neck malignancies over a decade. Our inclusion criteria encompassed 296 consecutive patients with locally advanced head and neck squamous cell carcinoma (HNSCC) - specifically stages III, IVA, and IVB, as classified per the American Joint Committee on Cancer, Seventh Edition (AJCC-7) guidelines [17].

Data collection

We collated a comprehensive dataset for each patient, including demographics, tumor characteristics, treatment regimens, dosimetric parameters, survival outcomes, and acute and chronic toxicities. Patients were treated with various radiotherapy regimens tailored to the extent of disease and therapeutic intent. These regimens ranged from definitive radical treatments, typically administered as 70Gy over 33 to 35 fractions or 66Gy over 33 fractions, to post-operative adjuvant courses of 60Gy over 30 fractions. Palliative treatments varied in dosage, primarily 30Gy delivered over 10 fractions or 20Gy over five fractions. The therapeutic modalities evolved during the study period, beginning with conventional intensity-modulated radiation therapy (cIMRT) in August 2009 and progressing to volumetric modulated arc therapy (VMAT) in its RapidArc iteration starting in 2012. For patients in the palliative care category, three-dimensional conformal radiation therapy (3D-CRT) was predominantly utilized.

Follow-up and outcome measures

We documented recurrence patterns, distinguishing between locoregional and distant metastatic progression. Locoregional control (LC) was stringently defined to capture the absence of tumor reemergence within the initial tumor site or regional lymphatics. Overall survival (OS) was another critical endpoint, calculated from the date of treatment completion to ensure consistency with the clinical endpoint of radiation effectiveness.

Toxicity assessment

Radiation-induced toxicities were systematically categorized by severity using the established Radiation Therapy Oncology Group (RTOG) Acute Radiation Morbidity Scoring Criteria [18], allowing for the standardized reporting of treatment-related side effects.

Statistical analysis

A detailed subgroup analysis was conducted, from which palliative cases and early 3D-CRT treatments were excluded to focus on the comparative effectiveness of VMAT and cIMRT in a curative setting. A total of 264 patients, 198 treated with VMAT and 66 with cIMRT, were included in the model-based analysis. Survival analyses, incorporating Kaplan-Meier curves, provided unadjusted survival probabilities. Multivariate analysis using Cox's proportional hazards models enabled us to adjust for various demographic and clinical factors, potentially confounding the relationship between treatment modality and survival outcomes. All statistical computations were performed using the R statistical software (R Foundation, Vienna, Austria), ensuring rigorous and reproducible analysis.

## Results

Among all 296 patients, the median follow-up for patients still alive was 42 months (range 6-107.3 months). The median age at diagnosis was 63 years old (range 26-89), 237 (80%) patients were male, 96 (32%) had a positive P16 histology, and 156 (53%) had at least two significant comorbidities. The most common primary tumor sites were the oropharynx (51.7%), larynx (14.5%), and hypopharynx (6.4%). The breakdown of stages based on AJCC 7th edition was as follows: stage III (19.9%), IVA (74.4%), and IVB (5.6%). Furthermore, 97% of patients received radiotherapy, 89% received 50Gy or higher radiation dose, including 66 (22%) cIMRT and 198 (67%) VMAT, 30% had surgery, and 61% had chemotherapy (platinum-based or cetuximab). Baseline patient characteristics are summarized in Table 1.

**Table 1:** Baseline patient demographics and clinical characteristics cIMRT - conventional intensity-modulated radiotherapy; VMAT - volumetric-modulated arc therapy

Characteristic	n	%
Age (years)	26-96	63 (median)
Sex		
Male	237	80%
Female	59	20%
Smoking history		
Smoker	114	39%
Non-smoker or unknown history	182	61%
P16 status available	124	42%
P16 positive	96	32%
Comorbidities		
With at least 1 major comorbidity	229	77%
With 2 or more major comorbidities	156	53%
Primary tumor site		
Nasopharynx	3	1.00%
Oropharynx	153	51.70%
Hypopharynx	19	6.40%
Larynx	43	14.50%
Nasal cavity and sinuses	4	1.40%
Parotid	8	2.70%
Skin	3	1.00%
Primary unknown	5	1.70%
Other	58	19.60%
Received EBRT	287	97%
50Gy or higher	264	89%
cIMRT	66	22%
VMAT	198	67%
Underwent planned surgery (primary and neck dissection)	89	30%
2nd salvage surgery	17	6%
Received chemotherapy	182	61%

Detailed analysis was performed on 264 consecutive patients who received radical doses by either VMAT or cIMRT. Marginal comparisons between the VMAT and cIMRT groups with respect to all prognostic factors are reported in Table 2. This analysis revealed no significant difference between the two treatments in these prognostic factors except for smoking.

**Table 2:** Analysis of baseline patient demographic data cIMRT - conventional intensity-modulated radiotherapy; VMAT - volumetric-modulated arc therapy; AJCC-7 - American Joint Committee on Cancer, Seventh Edition

Covariate		VMAT (N= 198)	IMRT (N= 66)	Parametric p-value*
Gender, (N (%))	Male	162 (81.82)	55 (83.33)	0.781
	Female	36 (18.18)	11 (16.67)	
Age at diagnosis (N)		198	66	0.494
Mean		63.08	62.02	
Median		63	59.5	
Stage (AJCC 7) (N (%))	III	40 (20.3)	12 (18.18)	0.928
	IVA	146 (74.11)	50 (75.76)	
	IVB	11 (5.58)	4 (6.06)	
Sites (N (%))	Nasopharynx	3 (1.52)	0 (0)	0.185
	Oropharynx	110 (55.56)	33 (50)	
	Hypopharynx	8 (4.04)	8 (12.12)	
	Larynx	30 (15.15)	8 (12.12)	
	Nasal cavity and sinuses	2 (1.01)	1 (1.52)	
	Parotid	7 (3.54)	0 (0)	
	Skin	2 (1.01)	0 (0)	
	Primary unknown	4 (2.02)	1 (1.52)	
	Other	32 (16.16)	15 (22.73)	
Smoking (N (%))	yes	60 (30.3)	40 (60.61)	< .001
	no	138 (69.7)	26 (39.39)	
Comorbidities (N (%))	No comorbidities	47 (23.74)	17 (25.76)	0.136
	Multiple	105 (53.03)	27 (40.91)	
Grade 3-4 toxicities (N (%))	yes	40 (20.73)	18 (27.69)	0.245
	no	153 (79.27)	47 (72.31)	
Permanent Xerostomia (N (%))	yes	27 (15.7)	3 (4.62)	0.022
	no	145 (84.3)	62 (95.38)	
Feeding tube (N (%))	yes	47 (24.23)	22 (33.85)	0.129
	no	147 (75.77)	43 (66.15)	

There was no treatment-related death, but 22.5% of the patients had grade 3-4 acute toxicity (RTOG Acute Radiation Morbidity Scoring Criteria). There was no significant difference between the two subgroups in grade 3-4 acute toxicity and the eventual need for a feeding tube (Table 2).

**Figure 1:**
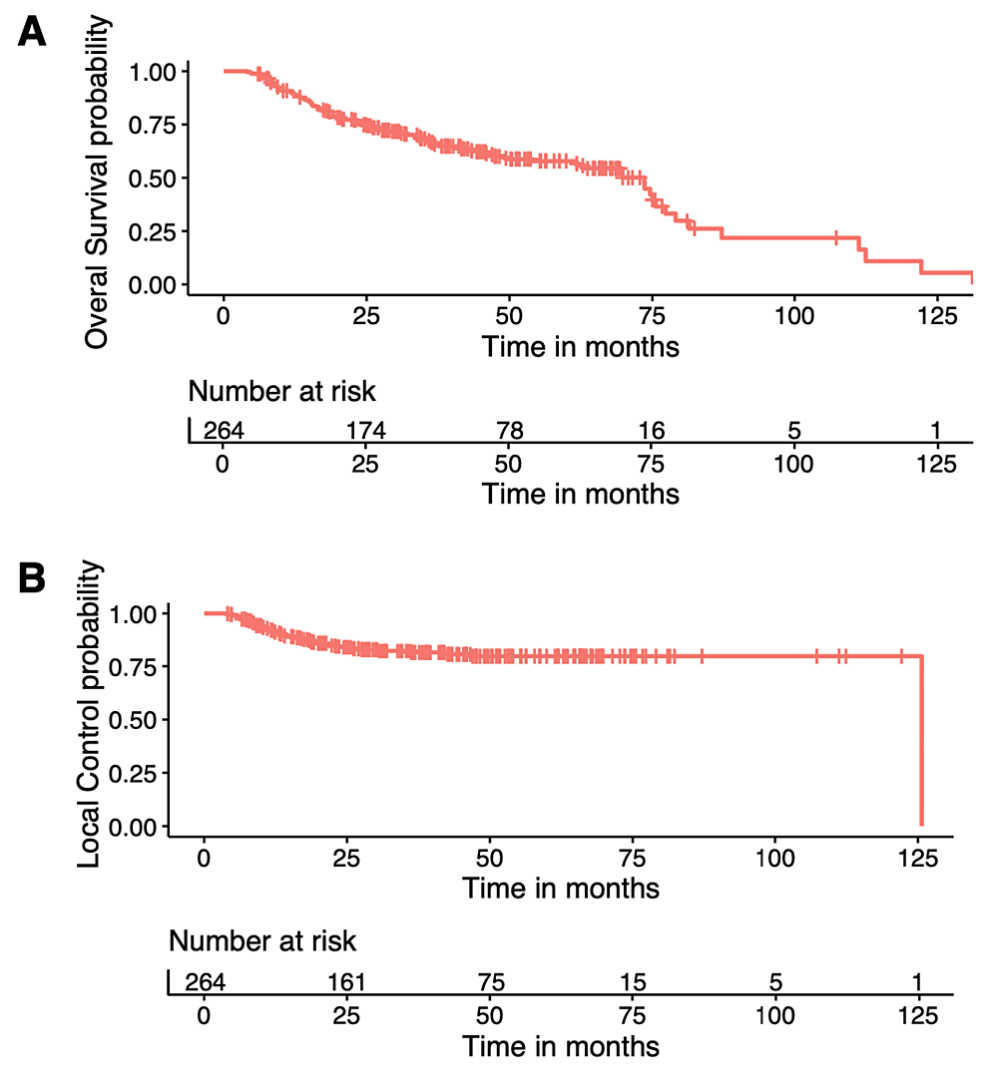
Overall survival (A) and local regional control (B) of the entire cohort

**Figure 2:**
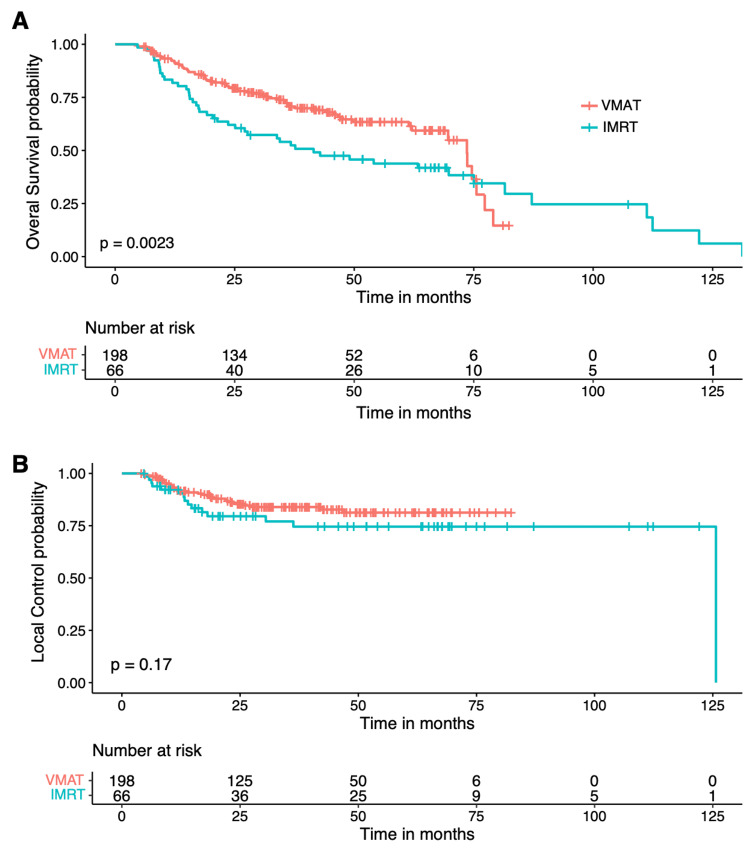
Overall survival (A) and local regional control (B) subdivided by treatment modality VMAT - volumetric-modulated arc therapy; IMRT - intensity-modulated radiotherapy

Using Kaplan-Meier Curves, the LC and OS of the entire cohort were 79.5% and 56.7% at five years, respectively (Figure 1). Further analysis demonstrated that treatment modality only significantly impacted five-year OS (VMAT 63.4% vs. cIMRT 43.8%, p=0.0023), but no significant difference was shown in the five-year LC (VMAT 81% vs. cIMRT 74.5%, p=0.17) (Figure 2).

Cox's model showed VMAT, stage, and age at the time of diagnosis were the only factors that significantly impacted OS, with a hazard ratio (HR) of 0.583 (95% CI 0.388-0.876, VMAT vs cIMRT), HR 0.38 (95% CI 0.155-0.933, stage III vs IVB), and HR 1.526 for every 10-year increase in age (95% CI 1.269-1.836), respectively. There was no significant difference in OS between stage III vs IVA or IVA vs. IVB. Age was the only factor that significantly impacted LC, with HR 1.4 (95% CI 1.046-1.874) for every 10 years increase in age (Tables 3 and 4).

**Table 3:** Hazard ratios for overall survival VMAT - volumetric-modulated arc therapy; IMRT - intensity-modulated radiotherapy

Factors	HR	95% confidence limits	
Treatment			
VMAT vs. IMRT	0.583	0.388	0.876
Stage			
III vs. IVA	0.636	0.371	1.091
III vs. IVB	0.380	0.155	0.933
IVA vs. IVB	0.597	0.274	1.301
Age at diagnosis			
10 years older	1.526	1.269	1.836

**Table 4:** Hazard ratios for local control VMAT - volumetric-modulated arc therapy; IMRT - intensity-modulated radiotherapy

Factors	HR	95% confidence limits	
Treatment			
VMAT vs. IMRT	0.581	0.306	1.104
Stages			
III vs. IVA	5.377	0.892	32.421
III vs. IVB	1.198	0.364	3.946
IVA vs. IVB	0.223	0.053	0.941
Age at diagnosis			
10 years older	1.400	1.046	1.874

## Discussion

In the last decade, IMRT has become more common in treating HNSCC. Several studies have demonstrated that IMRT has reduced toxicities, such as the decreased incidence of xerostomia post-radiation, compared to older technologies [10,19]. Radiation oncologists are able to prescribe a higher dose safely without worrying about severe toxicity to critical organs such as the spinal cord, which was difficult to achieve using 3D-CRT. VMAT, a newer technique of IMRT, has demonstrated reduced treatment planning and delivery time with improved dose homogeneity. It is a fast, safe, and accurate technique that uses lower monitor units (MU) by nearly 60% than cIMRT [12]. Still, few studies have directly compared the long-term clinical outcomes of VMAT to cIMRT.

Our present study is one of the first to evaluate the outcome of VMAT versus cIMRT for treating locally advanced HNSCC (stage III-IVB) between 2009 and 2019. Several previous studies have reported on the role of cIMRT and VMAT for specific HNSCC sites, such as the oropharynx, or explored these modalities for all stages of HNSCC. However, to date, few studies have focused on locally advanced HNSCC, which are associated with poorer outcomes. Nevertheless, in our analysis, the three-year LC and OS were 80% and 70%, and the five-year LC and OS were 79.5% and 56.7%, respectively, which is consistent with the values reported in the literature for IMRT treatment of locally advanced HNSCC [20-23].

In our study, treatment modality only significantly impacted five-year OS (VMAT 63.4% vs. cIMRT 43.8%, p=0. 0003), but not the five-year LC (VMAT 81% vs. cIMRT 74.5%, p=0.21) (Figure 2). Interestingly, Smet et al. also compared 78 cases of cIMRT and 79 cases of VMAT in locally advanced HNSCC, with almost all patients having stage III-IV disease, and found no significant differences in three-year OS (70.8 vs. 57.3%, p=0.219) or LC (84.9 vs. 72.7%, p=0.118) between the two groups [20]. This discrepancy in OS could be attributed to their equal (and also smaller) number of patients in the cIMRT and VMAT groups compared to our study, where 75% of the patients were treated with VMAT [20]. We have a long median follow-up of 42 months, making the five-year LC and OS analysis available. It is possible that the OS difference would become significant with longer follow-ups, as in our study. However, in the Smet et al. study, 80% of patients also received concurrent chemotherapy, as opposed to only 61% in our study (due to significant comorbidities), which could account for the observed differences. 

Limitations of the study

Considering the limitation of our study due to its retrospective nature, caution should be taken. The cIMRT subgroup was treated between 2009 and 2012, while the VMAT subgroup was treated between 2012 and 2019. There is selection bias due to the availability of more advanced radiation technology in recent years. Additionally, during this period, immunotherapeutic agents have improved recurrent and metastatic HNSCC patients' OS after treatment failure of standard dose concurrent chemoradiation [24]. Although none of our patients received salvage immunotherapy during the study period, improved supportive care might have contributed to better survival in recent years. It is also possible that the p16-positive oropharyngeal cancer patients were not equally distributed in the two subgroups, as we did not have routine p16 tests in the early years. Our institution started to use AJCC-8 staging after March 2019. It is impractical to convert all the staging of our cohort to this new version that considers p16 status as a very important prognostic factor, i.e., it has not been possible to obtain cell blocks to reassess these cases since 2009. P16-positive patients are known to have better OS compared to the p16-negative and cigarette smoking-related HNSCC.

Nonetheless, the difference in OS between VMAT and cIMRT with no difference in LC is unexpected. This is unusual, given that any form of radiotherapy is a local treatment. However, earlier studies did confirm that improved LC by treating early-stage breast cancer patients with postoperative radiation did translate into a survival benefit in the long term, even when systemic therapy was given [25]. Proportional gains might be larger because of more effective radiotherapy for breast cancer, which might be similar to HNSCC.

Additionally, there were no significant differences in acute or late toxicities between treatment modalities in our study (except for xerostomia), which could have indicated that the OS difference might be from the impact of the patient's other comorbidities. One such factor that could explain this anomaly is the significantly higher smoking rate in the cIMRT vs. VMAT cohort, 60.6% vs. 30.3%, thus, more likely to develop more future comorbidities associated with smoking and impact the OS. Due to the retrospective nature of our study, this could not have been circumvented.

Alternatively, this could be a statistical anomaly that other more extensive studies failed to show. Unfortunately, it is not practical to design a phase III multicenter randomized controlled trial to compare VMAT and cIMRT due to the obvious advantage of VMAT, such as patient comfort thanks to quicker delivery time (on average, three minutes for VMAT versus 10-15 minutes for cIMRT). Randomizing any patients into the cIMRT arm would be unethical should such a trial be planned in the future. A retrospective cohort study might be the only and best way to compare these two treatment modalities to locally advanced HNSCC.

## Conclusions

Our study presents evidence of VMAT's efficacy over cIMRT in enhancing overall survival for patients with locally advanced HNSCC. The results suggest that VMAT may improve long-term survival outcomes. Additionally, we identify a complex relationship between treatment modalities, patient characteristics, and treatment outcomes, emphasizing the importance of individualized treatment planning. The limitations of retrospective analyses, such as potential selection bias and evolving treatment protocols, were acknowledged. Nonetheless, the observed trend towards improved survival with VMAT supports its consideration in future clinical decision-making. This work contributes to the growing evidence for refining radiation therapy techniques and underscores the need for prospective research to enhance treatment efficacy against head and neck cancer.
